# The Minamata Convention and the phase down of dental amalgam

**DOI:** 10.2471/BLT.17.203141

**Published:** 2018-05-14

**Authors:** Julian Fisher, Benoit Varenne, Desiree Narvaez, Carolyn Vickers

**Affiliations:** a Department of Medical Informatics, Medizinische Hochschule Hannover, Hannover, Germany.; b Prevention of Noncommunicable Diseases Department, World Health Organization, avenue Appia 20, 1211 Geneva 27, Switzerland.; c Chemicals and Health Branch, United Nations Environment Programme, Geneva, Switzerland.; d Department of Public Health, Environmental and Social Determinants of Health, World Health Organization, Geneva, Switzerland.

Oral health is a neglected area of global health, although oral disease is one of the most common public health issues worldwide.^1^ Despite advances in modern dentistry, untreated dental caries in permanent teeth was reported as the most prevalent of the 328 conditions assessed in 2016 Global Burden of Disease Study.^2^ The restorative model for managing dental caries was developed in the 1900s, alongside dental amalgam as one of the restorative materials commonly used to treat dental caries. Together they still provide the backbone of oral health services in most countries today.

A shift away from the restorative model and the widespread use of dental amalgam was perhaps unimaginable even a decade ago, despite the World Health Organization (WHO) calling for oral health to be incorporated into policies for the integrated prevention and treatment of chronic noncommunicable and communicable diseases, and into maternal and child health policies.^3^

The Minamata Convention on Mercury (2013) is an international legally binding treaty that aims to protect the human health and the environment from anthropogenic emissions and releases of mercury and mercury compounds.^4^ The convention addresses mercury-added products, including dental amalgam, which is made of approximately 50% of elemental mercury by weight, and proposes nine measures to phase down the use of dental amalgam (Box 1). These measures show the interconnected and interdependent nature of phasing down dental amalgam, and reinforce the need for a multipronged approach as called for by WHO.

Box 1 **Minamata Convention, Annex A, Part II^4^**

**Nine measures to phase down the use of dental amalgam**

(i) Setting national objectives aiming at dental caries prevention and health promotion, thereby minimizing the need for dental restoration;

(ii) Setting national objectives aiming at minimizing its use;

(iii) Promoting the use of cost–effective and clinically effective mercury-free alternatives for dental restoration;

(iv) Promoting research and development of quality mercury-free materials for dental restoration;

(v) Encouraging representative professional organizations and dental schools to educate and train dental professionals and students on the use of mercury-free dental restoration alternatives and on promoting best management practices;

(vi) Discouraging insurance policies and programmes that favour dental amalgam use over mercury-free dental restoration;

(vii) Encouraging insurance policies and programmes that favour the use of quality alternatives to dental amalgam for dental restoration;

(viii) Restricting the use of dental amalgam to its encapsulated form;

(ix) Promoting the use of best environmental practices in dental facilities to reduce releases of mercury and mercury compounds to water and land.

The implementation of the convention and its provision for dental amalgam can catalyse the shift away from the restorative model of care and the use of mechanically retained filling materials, such as dental amalgam, towards preventive and minimal intervention dentistry that predominantly uses adhesive dental materials. Implementation will also provide an opportunity to strengthen oral health promotion and oral disease prevention within an integrated, people-centred model of health services (Box 1).

## Challenges of a phase down

A report from the United Nations Environment Programme revealed that mercury in dental use accounted globally for 270–341 metric tons in 2010, of which 70–100 tonnes (i.e. 20–30%) likely enters the solid waste stream.^5^ Disaggregated data on the supply and trade of bulk mercury for dental use as opposed to the encapsulated dental amalgam form is not available at a global level. More detailed information on global tracking of both forms would allow assessing the diversion of mercury destined for the dental sector to other sectors. Such tracking could support the development and implementation of national action plans to reduce, and where feasible, eliminate mercury use in artisanal and small-scale gold mining.^6^

Measuring and understanding the global impact and risk of the use of dental amalgam is critical for developing and coordinating multisectoral action needed to deliver an equitable and sustainable reduction of dental amalgam. The WHO report *Future use of materials for dental restoration*, which refers to the 1997 consensus statement on dental amalgam, identified a potential health risk to oral health personnel from mercury exposure exists if working conditions are not properly organized.^7^ These findings have been broadly supported by subsequent reports. For example, a report from the Scientific Committee on Emerging and Newly Identified Health Risks concluded that, current evidence does not preclude the use of either dental amalgam or alternative materials in dental restorative treatment.^8^

The ecotoxicology of dental amalgam waste released into waste water is well documented.^9^ While effective dental amalgam separator technology to capture dental amalgam waste exists and has been recommended as best management practice, it has not been universally implemented. At the same time WHO has highlighted the paucity of research evidence on the ecotoxicology of the mercury-free materials for dental restoration and has called for more quality studies and systematic reviews. Advocacy, social dialogue and policy dialogue are important instruments that can help to guide and catalyse sustainable investments, capacity building and policy action. Quality mercury-free materials are now selected and used as alternatives to dental amalgam, as a part of informed people-centred oral health care. However, it is important to distinguish between offering quality mercury-free materials as alternatives to dental amalgam and suggesting that one of these alternative materials could be a global replacement for dental amalgam. Large-scale systematic studies on the economic and social costs and benefits of quality mercury-free materials, such as resin-based composites or glass ionomer cements, have not yet been published. Lessons from Kenya, Uganda and United Republic of Tanzania in phasing down dental amalgam use note that low- and middle-income countries might face challenges in encouraging the use of such mercury-free materials. In rural settings, health facilities often lack a reliable supply of electricity and water, a necessary condition for the proper use of resin-based composites, which are more temperature sensitive and less tolerant to water during placement than dental amalgam.^10^

## Actions taken

Several countries have implemented actions to phase down the use of dental amalgam. Norway began a process of phasing down amalgam use in the late 1990s and since 2008 has implemented a general ban on mercury products that includes dental amalgam. As a part of this process, the Norwegian government introduced legislation that allowed time for the industry and for dentists to adapt to the new restrictions and guidelines.^11^

In the European Union, dental amalgam is the second largest use of mercury. From 1 July 2018, dental amalgam shall not be used in the treatment of deciduous teeth, children younger than 15 years and pregnant or breastfeeding women, except when strictly deemed necessary by the practitioner on the grounds of specific medical needs of the patient.^12^

## Needed policies and strategies

The phase down of dental amalgam can catalyse a profound change in dentistry through the convention and its provisions that focus attention on health needs and prioritize efficient provision of high-quality, affordable, integrated, community-based, people-centred primary and ambulatory care, paying special attention to underserved areas.

We suggest that a national coordination committee would facilitate efforts to phase down the use of dental amalgam. Such a committee could raise public awareness and support country-level communication strategies, which must be an early priority in the process. Coordinating stakeholders can help, guide and inform the process of change, contribute to understanding how to influence policy and facilitate taking action to strengthen oral health-care systems, particularly with respect to oral health-care financing.

Making progress towards universal health coverage (UHC) requires governments to have mechanisms to effectively manage oral health workforce planning, and to commit to mobilize and sustain adequate public funding for oral health, including budgetary resources for phasing down dental amalgam.

National education plans for the oral health workforce in support of UHC should be aligned with national oral health plans. Strategies and programmes for the prevention and control of noncommunicable diseases should consider the role and contribution of the oral health workforce. At the same time, evidence-based reviews could encourage and support insurance companies to examine policy and programme options that favour a shift to quality mercury-free materials for dental restoration, including materials that re-mineralize tooth substance.

We believe that efforts to phase down the use of amalgam should be directed towards a multipronged approach that combines waste management, knowledge management and health systems strengthening. Table 1 proposes a set of strategic interventions aligned with the nine measures set out in Annex A of the Minamata Convention on Mercury.^4^ This would help addressing the economic, social and sustainability concerns in a balanced and holistic manner.^13^

**Table 1:** Strategic interventions aligned with the nine measures set out in Annex A Part II of the Minamata Convention^4^

Strategic interventions	Corresponding measures, Annex A Part II
**Waste management**	
Implementing a strategic approach to integrated chemicals management, one that combines infection-control measures, best management practices and an environmentally sound lifecycle management of dental amalgam waste and quality mercury-free dental materials waste	Measures ii, ix
Making the use of dental amalgam in an encapsulated form and amalgam separators mandatory	Measures viii, ix
**Knowledge management**	
Carrying out a situation assessment and inventory of trade, supply, regulation and use of dental amalgam and quality mercury-free materials for dental restoration, to develop clear time-bound targets. The assessment will guide and inform a baseline against which progress in achieving the targets can and will be measured	Measures ii, iii
Developing and documenting good practice examples and demonstrating the feasibility of voluntary implementation of the phase down of the use of dental amalgam	Measures i, ii
Raising public awareness and supporting country-level communication strategies and programmes based on the results of the situation assessment and documentation of voluntary implementation	Measures i, ii, iii, viii, ix,
**Health system strengthening as an integral part of an equitable and sustainable reduction of the use of dental amalgam**	
Integrating strategies to phase down dental amalgam as part of the policy of prevention and control of noncommunicable disease and addressing the social determinants of health	Measure i
Improving the affordability of mercury-free materials for dental restoration in the essential list of medicine, products and medical devices and equipment managed by the national and regional authority in charge of purchasing regional and national pharmaceuticals	Measure iii
Researching and developing quality, affordable and safe alternatives to dental amalgam whose waste can be controlled in an efficient and effective manner in all health-care settings	Measure iv
Reorientation of the oral health workforce education agenda towards greater social accountability, and of educational curricula to meet local community needs. Increasing national capacity of oral health professionals towards preventive and non-operative minimally invasive dental care by developing training materials and resources	Measures i, v
Encourage and support insurance companies to examine policy and programme options that favour a shift to quality mercury-free materials for dental restoration, including materials that re-mineralise tooth substance and inhibit dentine demineralisation	Measures vi, vii

^a^ The measures are explained in Box 1.

We are in a period of transition from a conventional model of restorative dentistry, one largely based on the use of dental amalgam, to an oral health model oriented towards health promotion and integrated disease prevention. The phase down of the use of dental amalgam can become a catalyst to renew and revitalize dentistry and tackle the health, social and economic burden of oral disease by prioritizing oral health as part of the global health agenda.
